# Prevalence and associated risk factors of low back pain among bank employees in Pokhara, Nepal

**DOI:** 10.1186/s12889-026-28491-x

**Published:** 2026-07-08

**Authors:** Niraj Nagarkoti, Dhirendra Nath, Bikram Singh Dhami, Basant Kumar Dhami, Rajendra Lamichhane

**Affiliations:** 1 School of Health and Allied Sciences, Pokhara University, Kaski, Nepal; 2 Health Directorate, Ministry of Social Development, Doti, Sudurpaschim Province Nepal; 3 Kathmandu Institute of Child Health (KIOCH), Kathmandu, Nepal; 4 School of Development and Social Engineering, Pokhara University, Kaski, Nepal

**Keywords:** Banker employees, Bank, Low back pain, Musculoskeletal Disorder, Nepal

## Abstract

**Background:**

Low Back Pain (LBP) is a prevalent musculoskeletal condition among office professionals, particularly bankers, who often experience prolonged sitting and poor ergonomic conditions. Globally, LBP is the leading cause of disability for both sexes and across all age groups. However, research on occupational and psychosocial determinants among banking professionals in Nepal remains limited. This study aims to assess the prevalence and associated risk factors of LBP among bankers in Pokhara, Nepal.

**Methods:**

A cross-sectional study was conducted from January to February 2023 among 334 systematically selected bank workers from 27 banks within Pokhara Metropolitan, Kaski District. Participants with at least three months of work experience were included in the study. Data were collected through face-to-face interviews using a semi-structured questionnaire. LBP-related information was assessed using the Extended Nordic Musculoskeletal Questionnaire (NMQ-E). Stepwise logistic regression modeling was employed to identify the associated factors. Adjusted odds ratios (AORs) with 95% confidence intervals (CIs) were calculated, and *p*-values < 0.05 were considered statistically significant. Furthermore, the random forest technique was employed to identify the major variables.

**Results:**

Of the 334 employees, on average, they were 31 years old, and more than half (55.1%) were female. The one-year prevalence of LBP was found to be 56.3% (95% CI: 51.2–61.7). LBP was positively associated with female employees (AOR: 2.62, 95% CI: 1.61–4.27), employees having higher education (AOR: 3.91, 95% CI: 1.22–12.45), and employees experiencing work-related stress (AOR: 3.68, 95% CI: 3.68, 95% CI: 2.16–6.29). While married employees (AOR: 0.36, 95% CI: 0.21–0.59) and private bank employees (AOR: 0.31, 95% CI: 0.15–0.65) were less likely to have LBP. The major variables explaining LBP, as identified by the random forest technique, in order, are: work-related stress, marital status, gender, type of bank, and education level.

**Conclusions:**

The prevalence of LBP among bank workers in Pokhara was 56.3%, indicating a substantial burden of musculoskeletal conditions among bank workers. Preventive measures to address work-related stress, along with consideration of gender and marital status while designing ergonomic interventions, are crucial for lowering LBP among bank employees.

## Introduction

Low Back Pain (LBP), a common problem faced by people of all ages [1], is defined as the pain experienced between the lower rib margins and the buttock creases [2]. The pain is often accompanied by discomfort in one or both legs, and some individuals with LBP also experience neurological symptoms in their lower limbs [1]. People who experience LBP frequently have concurrent pain in other body parts and higher general physical and mental health problems than those without LBP [3]. The impacts of LBP are broad and cross-sectoral; disability, diminished ability to engage in family, social, and work roles, and poses an economic burden to the families, societies, and countries [4]. Similarly, chronic LBP is associated with adverse psychosocial impacts [5], comorbidities, and higher mortality [6], and poor health-related quality of life [7]. Additionally, people with LBP were more likely to experience poverty, early exit from the workforce, and collect lower retirement money [8, 9].

Globally, in 2020, about 1 in 13 people had LBP (age-standardized point prevalence of 7.5%), affecting 619 million people, representing an increase of 60% in cases compared to the 1990 estimates [10]. According to the Global Burden of Disease (GBD) study, the prevalence rate of LBP rises at age 15 and peaks at age 85 [10]. Moreover, LBP is the leading cause of disability worldwide for both sexes and across all age groups, accounting for 8% of all Years Lived with Disability (YLD) [10]. From 1990 to 2020, a 59% increase in YLD was attributed to LBP, with the highest increase in low and middle-income countries due to population growth and aging [10].

In the South East Asia Region (SEAR), the reported age-standardized prevalence rate of LBP is estimated at 5880 per 100,000 population, with 40.7 million cases [10]. The prevalence of LBP varies significantly among occupational groups in South Asia. Among bank employees, the annual prevalence was reported to be 62.5% in Bhopal, India [11], 51.8% in Tamil Nadu, India [12], and 52.4% in Pakistan [13], while the one-month prevalence in Dhaka, Bangladesh, was 36.6% [14]. Similarly, the annual prevalence of LBP was 42.7% among farmers, rickshaw pullers, and office workers in Bangladesh [15], 44% among laparoscopic and general surgeons in Pakistan [16], 43% among rubber tappers in Sri Lanka [17], and the 4-month prevalence among drivers of Sri Lanka was 15.5% [18]. In the general population, the one-year prevalence of LBP was 18.6% in Bangladesh [19], 48% in Northern India [20], while the 2-year prevalence among older adults in India is 32.6% [21].

In Nepal, the annual prevalence of LBP ranges from 24.7% among orthopedic patients [22], 43.8% among motor bike riders [23], 52% among construction workers [24], 65% among nurses [25], to 71% among the general population [26], while one month prevalence among textile workers was 35% [27]. Despite the high burden, there is limited research on LBP, particularly focusing on bank employees in Nepal.

Earlier literature highlighted demographic characteristics – such as age [14, 27–31], gender [12, 24, 27–29, 32], educational level [30, 31, 33], marital status [26, 30], behavioral characteristics – such as smoking [10, 30], alcohol consumption [34], physical activity [14, 28, 31, 32, 35], sleep pattern [24, 28], Body Mass Index (BMI) [10, 14, 15], and work related characteristics – such as work related stress [12, 31, 32], type of chair [32, 36], lifting objects at work [32], seating position at work [31, 36], daily working hours [14, 31], and work time break [31, 36] associated with LBP among various population including bank employees.

The occupational transition from traditional agricultural practices to sedentary office work has augmented the risk of LBP among working professionals in Nepal, due to prolonged sitting, bending, or twisting seating positions, repetitive motions, and poor ergonomic conditions at the workplace. In addition, Nepal faces major challenges in reducing the burden of LBP due to an undersupply of health professionals, overuse of ineffective healthcare services, and high out-of-pocket expenditure [37]. Identifying and evaluating the importance of factors affecting LBP among bank employees, therefore, will be pivotal for formulating context-specific programs and strategies to address LBP. Hence, this study aims to assess the prevalence of LBP among bankers and its associated factors in Pokhara, Nepal.

## Methods

### Study design and setting

The cross-sectional study was conducted in Pokhara, the second largest city after Kathmandu in Nepal, and it is situated 200 KM west of Kathmandu [38]. The area of Pokhara is 464.24 km², and the total population of 513 504 [39]. Pokhara is a rapidly developing commercial and urban area with many banks and financial institutions. According to the Bank and Financial Institution Act of Nepal (BAFIA) 2006, banks and financial institutions are classified into four different groups: Group A: Commercial Banks, Group B: Development Banks, Group C: Financial companies, and Group D: Micro credit development banks [40]. Our study included participants from Group A and Group B; however, Group C and Group D were not included in this study as they primarily serve smaller, more localized markets and have different organizational structures and employee workloads. Moreover, the participants’ availability and scale of operation in Group C and Group D did not align with the scope of our study.

### Study population and selection criteria

Bank employees were the study population in this study. Full-time bank employees who maintained a regular office past three months were included in this study [41], while female employees who had a baby younger than six months were excluded [14].

### Sample size and sampling technique

The sample size was calculated using the Cochran’s formula for a single proportion, i.e., n_0_ = Z^2^ p(1-p)/d^2^ [42]. Based on the existing literature, there is no specific study conducted among bank employees in Nepal to assess the prevalence of LBP; we used a proportion of 0.5.The initial sample size obtained was 384, considering the proportion of low back pain (p): 0.5, margin of error (d): 5% and confidence interval: 95%.

Since the total number of bank employees working in class ‘A’ and class ‘B’ banks, as provided by the provincial office of Nepal Rastra Bank (NRB), was 2588, we used a finite population correction using the formula: n = n_0_/ [1+(n_0_-1)/N]. Finally, considering these factors, the calculated sample size was 334.

Multistage systematic random sampling was used to select the study participants. In the first stage, the proportion of Group A and Group B banks was calculated; of the total employees, 55% were from Group A banks, and the remaining 45% were from Group B banks. Then, the proportion of the 15 Group A banks and the 12 Group B banks was computed using the total employees of Group A and Group B banks, respectively. Finally, participants from each bank were selected through systematic random sampling.

### Data collection procedure

A semi-structured questionnaire was used to collect data from participants through face-to-face interviews. The questionnaire consists of four sections: socio-demographic characteristics, behavioral characteristics, work-related information, and the Extended Nordic Musculoskeletal Questionnaire (NMQ-E). The extended NMQ-E measures LBP among bank employees, including specific questions focusing on the past year, four weeks, and lifetime prevalence of LBP. This consists of 11 dichotomous (Yes/No) questions related to the frequency of LBP, impact, severity, and health-seeking due to trouble related to LBP [43]. For this study, the extended Nordic questionnaire was adopted from a study conducted among construction workers in Nepal [24]. The researchers carefully examined every question to ensure simplicity, clarity, and understandability. The first author (NN) collected data using a paper-based questionnaire from 20/01/2023 to 17/02/2023.

### Anthropometric measurement

The height of the participants was measured with a measuring tape, and a portable digital weighing machine was used to measure the weight.

### Study variables

#### Outcome variable

##### Low back pain

In this study, an individual experiencing low back pain (LBP) was defined as someone who reported a sensation of ache, pain, or discomfort localized between the lower rib margin and the upper gluteal fold within 12 months. The variable was assessed dichotomously, with responses categorized as either ‘yes or no’ [32].

### Independent variables

#### Sociodemographic information

These included: age, gender (male, female), marital status (unmarried, married, separated), and educational status (basic level, secondary level, bachelor’s, master’s, and above). The age of the participants was recorded as a continuous variable (in completed years); however, in analysis, age was treated as dichotomous (< 30 years and ≥ 30 years) based on the median value and sample distribution.

#### Behavioral information

Behavioral information consists of: smoking habit (current smoker, past smoker, never smoke), alcohol consumption (current drunker, past drunker, never drink), physical activity (sufficient, insufficient), sleep pattern (normal, abnormal), and Body Mass Index (BMI) (underweight, normal, overweight, obese).

#####  Smoking habit

Smoking habit was categorized into two groups: ‘never and current/previous’. Current smokers are defined as individuals who reported smoking any form of tobacco product within the past 30 days. Previous smokers are those who reported regular smoking at some point in their lifetime but were not smoking at the time of the survey [14].

##### Physical activity

Sufficient physical activity was defined as the participants who engaged in any kind of physical activity or sports, including walking for at least 150 min per week; otherwise, they were classified as having insufficient physical activity [44].

#####  Sleep pattern

The sleep hour of the participants was calculated based on their bedtime at night and wake-up time in the morning. Then the obtained sleep hour is categorized into two groups: normal and abnormal. Sleep hour is considered normal if a person sleeps for 7–8 h; and if a person sleeps for less than 6 h or more than 8 h, the sleep pattern is considered abnormal [45].

#####  Body Mass Index (BMI)

Body Mass Index is defined as the weight in kilograms divided by the square of the height in meters (kg/m^2^) and is classified as: Underweight: <18.5, Normal: 18.5-24.99, Overweight: 25.0-29.99, and Obese: *≥* 30.00 [46].

#### Work-related information

Work-related information consists of; type of bank (government, private), self-reported work-related stress (yes, no), regular use of computer at workplace (yes, no), type of computer used (desktop, laptop), daily computer usage (in hours), years of computer use, type of chair (fixed, moveable), having a chair with armrest (yes, no), seating position at work (back straight, back bent, back twisted), daily seating hours, lifting objects at work (yes, no), break time (no break, sometimes, frequent break).

##### Work-related stress

It was assessed by asking a question; Did you feel stressed at your workplace? The response was recorded as a dichotomous variable, with options yes or no.

### Reliability and validity of the study

A validated extended NMQ-E questionnaire was used to assess the LBP among bank employees. Previous studies have used this tool in Nepal to assess LBP among employees of various occupations [24, 47]. Initially, the questionnaire was developed in the English language, and then it was translated into the Nepali language and then back-translated into English to ensure its consistency. Before conducting actual data collection, pretesting of the questionnaire among 10% (*n* = 34) of the sample size was done; the sequence of questions was rearranged and language was refined to ensure clarity and readability. Moreover, during the data collection period, questionnaires were checked for completeness and consistency at the end of each day.

### Data management and analysis

Data were entered and managed in Epidata version 3.1 and then transferred to Statistical Package for Social Sciences (SPSS) version 21 for analysis. In the descriptive analysis, categorical variables were presented as frequency and percentage, while continuous variables were summarized as mean, median, Standard Deviation (SD), and Inter Quartile Range (IQR) based on data distribution.

Stepwise multivariate binary logistic regression analysis was conducted using the forward selection method based on the Wald criterion to determine predictors of LBP. Initially, unadjusted odds ratios (UOR) were calculated for all variables of interest - sociodemographic characteristics, behavioral and lifestyle characteristics, and work-related factors. Two distinct models were constructed for further analysis. Model 1 included variables with a *p* < 0.25, while Model 2 focused on variables with a *p* < 0.05 identified through the UOR calculation. During the stepwise multivariate analysis, the criteria for inclusion were set at a significance threshold of *p* < 0.05. Variables were retained in the models if their *p*-values remained below 0.10, allowing for a more flexible approach to variable selection without prematurely discarding significant predictors [48]. The analysis resulted in six steps for Model 1 and five for Model 2. The variables retained in Model 1 were: gender, marital status, education level, work-related stress, type of bank (public vs. private), and smoking status. Consequently, in Model 2, gender, marital status, education level, work-related stress, and type of bank were retained.

The Hosmer-Lemeshow test was used to assess the goodness of fit for the model applied, with a p-value greater than 0.05 indicating an acceptable fit. To further evaluate model fit, we computed the Log Likelihood Ratio (LLR), Akaike Information Criterion (AIC), and Bayesian Information Criterion (BIC) for Model 1 and Model 2. The LLR for Model 1 and Model 2 were 393.36 and 400.22, respectively, suggesting that Model 2 provides a better fit to the observed data. Additionally, the AIC values for Model 1 and Model 2 were 92.018 and 73.075, respectively, while the corresponding BIC values were 108.696 and 95.942. The lower AIC and BIC value of Model 2 shows that it provides a better balance between goodness of fit and model complexity compared to Model 1 [48]. Based on these results, Model 2 with the highest LLR, lowest AIC, and Lowest BIC, was considered the best fit model.

Furthermore, a random forest classification model was used to evaluate the importance of variables affecting LBP. The random forest package in R, having two variables per split (mtry: 2) and 500 trees (ntree: 500), was used to construct a random forest model. Likewise, Mean Decrease Accuracy (MDA) and Mean Decrease Gini (MDG) were used to evaluate the significance of each variable to provide their predictive power. Coupled with a Receiver Operating Characteristic (ROC) Curve analysis, an out-of-bag (OOB) error rate was calculated to ensure sound model evaluation.

## Results

### Descriptive characteristics of the participants

Table 1 shows the sociodemographic, behavioral, and work-related characteristics of the participants. More than half (56.0%) of the participants were under 30 years of age, and 55.1% of them were female. Similarly, slightly less than half (47.6%) of the participants have a master’s and above degree while about three-fifths of them were married (Table 1).

**Table 1:** Socio-demographic and work-related characteristics of bank employees (*n* = 334) in Pokhara, Nepal

Variables	Frequency (*n*)	Percentage (%)
Sociodemographic information		
Age (Mean *±* SD: 31.58 *±* 5.47 years)		
*≤* 30 years	187	56.0
31–40 Years	122	36.5
*≥* 41 years	25	7.5
Gender		
Male	151	45.20
Female	183	54.80
Educational Status		
Secondary level	17	6.3
Bachelor	158	47.3
Master and above	159	47.6
Marital status		
Unmarried/Separated	138	41.0
Married	196	59.0
Behavioral information		
Smoking habit		
Current	21	6.3
Past smoker	38	11.4
Never	275	82.3
Alcohol consumption		
Current user	88	26.3
Past alcohol use	38	11.4
Never	208	62.3
Physical activity		
Sufficient	131	39.2
Insufficient	203	60.8
Sleep pattern		
Abnormal	27	8.1
Normal Sleep	307	91.9
Body Mass Index (BMI)		
Underweight	9	2.7
Normal	192	57.5
Overweight	118	35.3
Obese	15	4.5
Work-related characteristics		
Type of bank		
Government	48	14.4
Private	286	85.6
Work-related Stress		
Yes	228	68.3
No	106	31.7
Regular use of computer at workplace		
Yes	333	99.7
No	1	0.3
Type of computer used		
Desktop	297	88.9
Laptop	37	11.1
Daily computer usage (hours)		
Median:		
< 4 h	115	34.4
*≥* 4 h	219	65.6
Years of computer use		
*≤* 1 years	22	6.6
1 to 5 years	103	30.8
More than 5 years	209	62.6
Type of chair		
Fixed chair	31	9.3
Movable chair	303	90.7
Having a chair with arm rest		
Yes	257	76.9
No	77	23.1
Seating position at work		
Back straight	198	59.3
Back bent	111	33.2
Back twisted	25	7.5
Daily seating hours		
*≤* 6 h	145	43.4
> 6 h	189	56.6
Lifting objects at work		
Yes	103	30.8
No	231	69.2
Break time		
Having no break	27	8.1
Sometime	249	74.6
Frequent breaks	58	17.4

In terms of behavioral information, (6.3%) of them were current smokers, while about one-fourth (26.3%) were current alcohol users. Furthermore, only two-fifths (39.2%) of bank employees have sufficient physical activity, and the majority (91.9%) of them have a normal sleep pattern. Additionally, about two in five employees were overweight/obese. (Table 1)

Similarly, in work-related characteristics, the majority (85.6%) of the employees were from private banks, and over two-thirds (68.3%) of them reported work-related stress. Nearly almost (99.7%) of the participants used a computer, approximately two-thirds (65.6%) used a computer for more than 4 h per day, and the majority of them had been using computers for more than 5 years. Most participants (90.7%) had used a movable chair, and 76.9% had chairs with armrests. Regarding seating position, 59.3% kept their backs straight, while about a third (33.2%) had a bent back during work. Just over half (56.6%) of participants sat for more than 6 h per day compared to those who sat for less than 6 h (43,4%). Approximately seven in ten participants did not lift objects as part of their work. Regarding break times, almost three-fourths (74.6%) took breaks sometimes, while 17.4% took frequent breaks, and only a few 98.1%) did not take breaks at all (Table 1).

### Prevalence of low back pain (LBP)

**Fig. 1:**
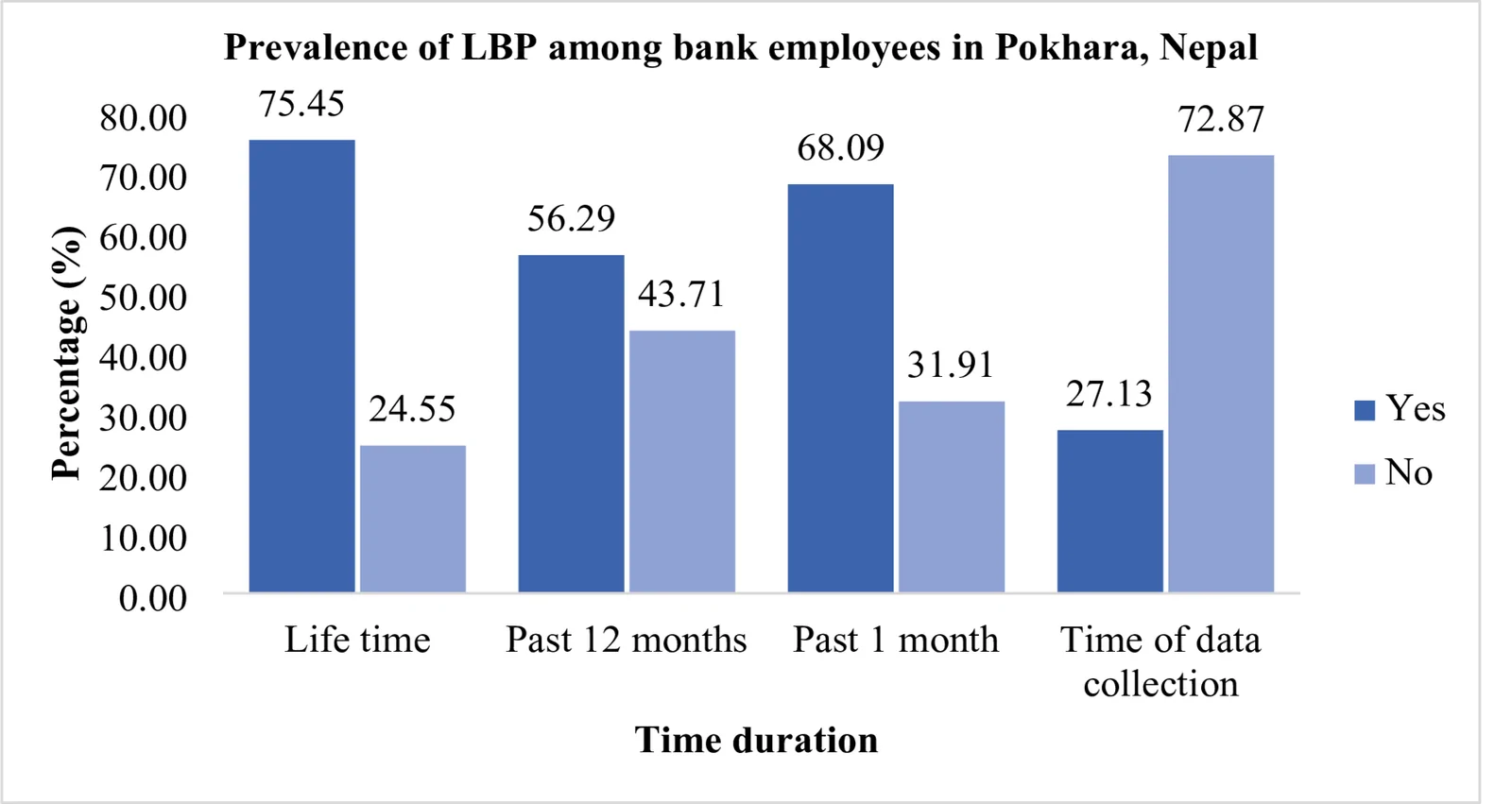
Prevalence of low back pain (LBP) among bank employees in Pokhara, Nepal

The figure demonstrates that a significant proportion of study participants have experienced low back pain (LBP), with the lifetime prevalence of LBP being 75.4%, and 56.3% experienced LBP in the last year. Among them, 68.1% of the participants reported having trouble in the last 4 weeks. Moreover, on the day of the survey, just over a quarter (27.1%) of participants reported having discomfort/trouble. (Fig. 1)

### Factors associated with LBP among bank employees

Table 2 shows the factors associated with LBP among bank employees. From stepwise regression analysis, female employees (AOR: 2.62, 95% CI: 1.61–4.27) were far more likely to have LBP than males. Similarly, participants with a bachelor’s or above educational level (AOR: 3.91, 95% CI: 1.22–12.45) were nearly four times more likely to have LBP than those having education up to secondary level. Additionally, participants who had work-related stress (AOR: 3.68, 95% CI: 2.16–6.29) were about 3.5 times more likely to report LBP than their counterparts. Meanwhile, married employees (AOR: 0.36, 95% CI: 0.21–0.59) had lower odds of LBP than single employees (separated/divorced). Likewise, private bank employees (AOR: 0.31, 95% CI: 0.15–0.65) were less likely to report LBP than government bank employees (Table 2).

**Table 2:** Factors associated with Low Back Pain among bank employees in Pokhara, Nepal

Variables	Categories	Unadjusted OR		Model 1	Model 2
		COR (95% CI)	*p*-value	AOR (95%CI)	AOR (95% CI)
Gender	Male	1.00	**-**	1.00	1.00
	Female	1.94 (1.25–3.02) *	**0.003**	3.37 (1.97–5.76) ***	2.62 (1.61–4.27) ***
Age	*≤* 30 years	1.00	**-**		
	> 30 years	0.56 (0.36–0.87) *	**0.009**		
Body Mass Index (BMI)	Healthy weight	1.00	-		
	Unhealthy weight	1.08 (0.69–1.69)	0.74		
Marital status	Single (Separated/Divorced)	1.00	**-**	1.00	1.00
	Married	0.48 (0.31–0.75) **	**0.001**	0.36 (0.21–0.60) ***	0.36 (0.21–0.59) ***
Education status	Secondary level or below	1.00		1.00	1.00
	Bachelor and/or higher	3.28 (1.13–9.53) *	**0.029**	4.01 (1.27–12.68) **	3.91 (1.22–12.45) *
Type of bank	Government bank	1.00		1.00	1.00
	Private bank	0.48 (0.25–0.93) *	**0.031**	0.29 (0.14–0.62) ***	0.31 (0.15–0.65) *
Work-related stress	No	1.00		1.00	1.00
	Yes	2.42 (1.51–3.87) ***	**< 0.001`**	3.77 (2.19–6.47) ***	3.68 (2.16–6.29) ***
Smoking status	Never	1.00		1.00	
	Smoker at some point in time	1.51 (0.84–2.70)	0.17	2.46 (1.23–4.89) **	
Physical activity	Sufficient	1.00			
	Insufficient	1.70 (1.08–2.67) *	**0.021**		
Sleep pattern	Normal	1.00			
	Abnormal	0.51 (0.23–1.12)	0.094		
Type of computer	Desktop	1.00			
	Laptop	1.16 (0.58–2.32)	0.68		
Computer use per day (hrs)	< 4 h	1.00			
	*≥* 4 h	0.75 (0.48–1.19)	0.22		
Duration of computer use	*≤* 5 years	1.00			
	> 5 years	0.60 (0.38–0.95) *	**0.029**		
Type of chair	Movable chair	1.00			
	Fixed chair	1.46 (0.68–3.16)	0.33		
Chair with armrest	Yes	1.00			
	No	0.85 (0.51–1.42)	0.54		
Seating Position	Back Straight	1.00			
	Back bent	1.80 (1.15–2.81) **	**0.010**		
Time spent sitting (per day)	*≤* 6 h	1.00			
	> 6 h	0.89 (0.57–1.38)	0.60		
Nature of job	Didn’t require lifting objects	1.00			
	Required lifting objects	0.67 (0.42–1.07)	0.096		
Breaks during work hours	Frequent break	1.00			
	Sometimes	0.99 (0.39–2.47)	0.97		
	No breaks	1.87 (1.05–3.32) *	**0.034**		
-2 Log likelihood ratio				393.36	400.22
Cox and Snell R^2^				0.175	0.158
Nagelkereke R^2^				0.235	0.212
AIC				92.018	73.075
BIC				118.696	95.942

Model-1 includes variables with *p*-values less than 0.25 in the unadjusted model and model-2 includes variables with *p*-values less than 0.05 in the unadjusted model

*COR* Crude Odds Ratio, *AOR* Adjusted Odds Ratio, *AIC* Akaike Information Criterion, *BIC* Bayesian Information Criterion

*Statistically significant at *p*-value < 0.05, **Statistically significant at *p*-value < 0.01, and ***Statistically significant at *p*-value < 0.001

### Variable importance based on random forest model

**Fig. 2:**
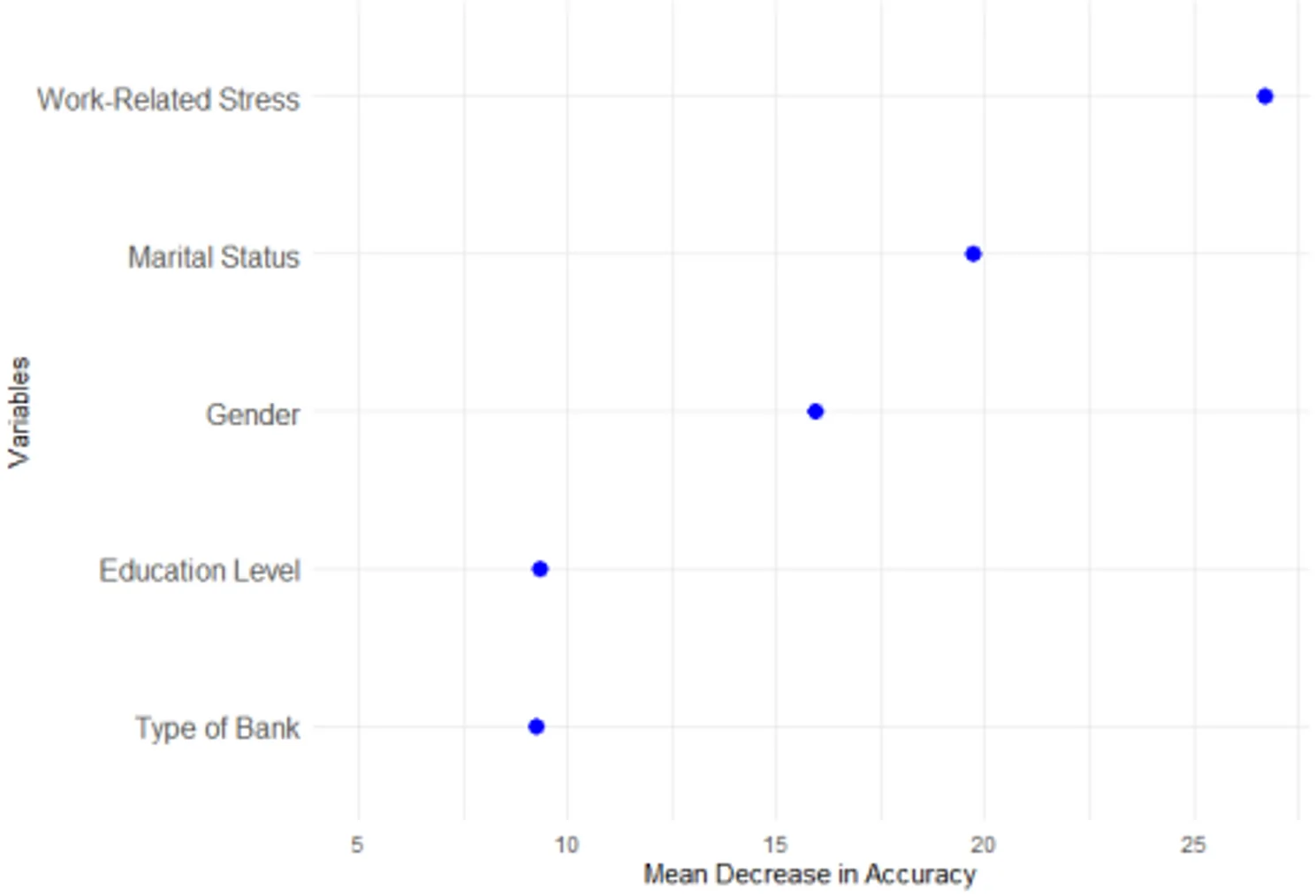
Forest plot of variable importance (Mean Decrease in Accuracy)

**Fig. 3:**
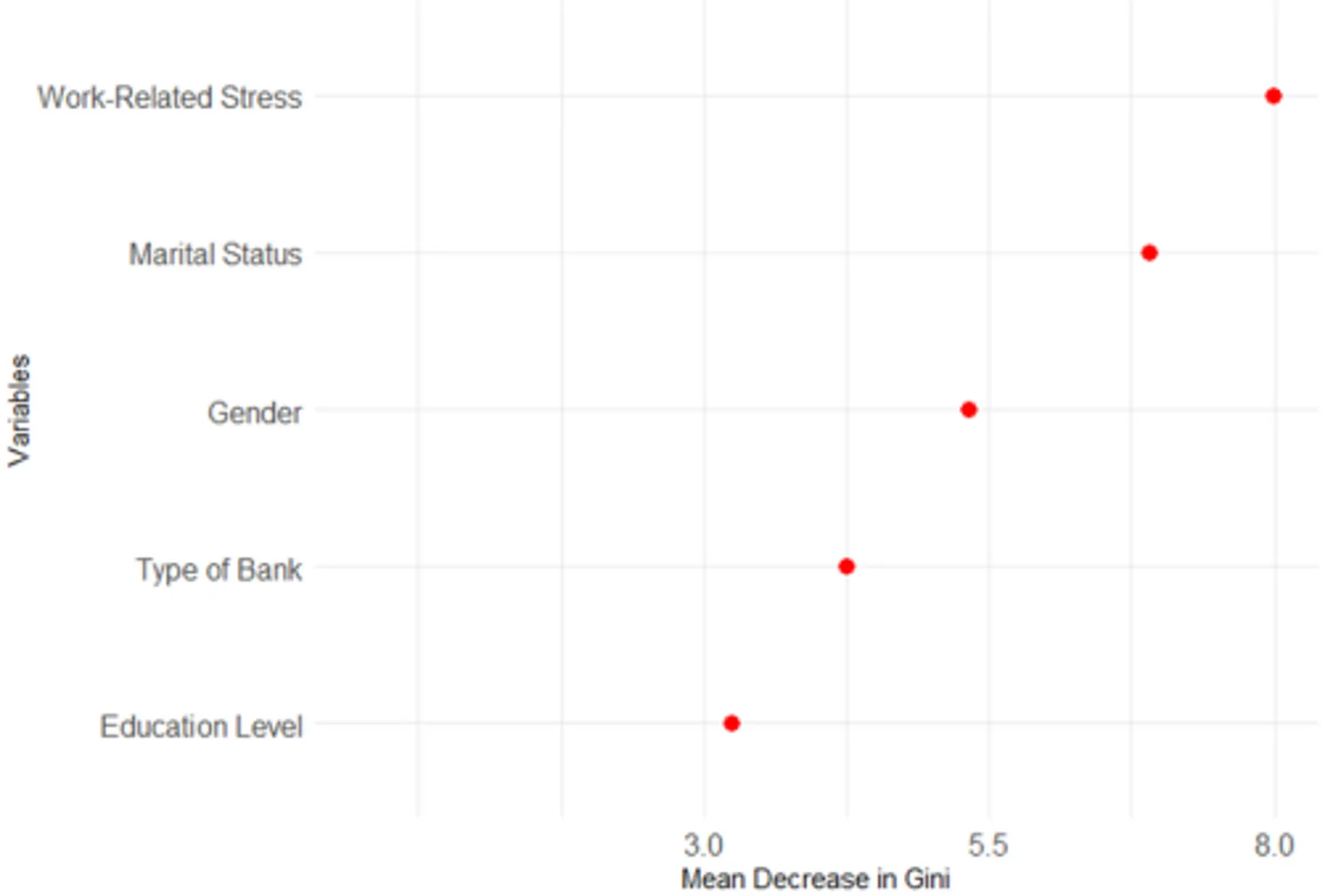
Forest plot of variable importance (Mean Decrease in Gini)

The assessment of variable importance identified work-related stress as the most influential predictor, with a Mean Decrease in Accuracy (MDA) value of 26.72, indicating a substantial impact on classification accuracy. Similarly, gender (MDA: 19.72) and marital status (MDA: 15.96) were also found to be significant predictors; however, type of bank and educational level were of less significance (Fig. 2). The finding of MDA was also corroborated by the result of Mean Decrease in Gini (MDG), where work-related stress was found to be the most influential predictor (MDG: 7.99), and marital status (MDG: 6.90), and gender (MDG: 5.32) were also notable variables. While the type of bank (MDG: 4.25) and educational level (MDG: 3.24) have relatively low MDG values, indicating their lower impact on the classification model. (Fig. 3)

## Discussion

This study determined the LBP and its associated factors among bank employees in Pokhara, Nepal. The findings revealed a substantial proportion of LBP among bank employees, with a lifetime prevalence of 74.5% (95% CI: 71.0-80.2) and a one-year prevalence of 56.3% (95% CI: 51.2–61.7). These results demonstrate that low back pain is one of the common musculoskeletal conditions among bank employees in our study area. Furthermore, this study identified several factors associated with higher odds of LBP. Female employees, married participants, those with higher educational levels, and employees who reported perceived stress at the workplace had higher odds of experiencing LBP. Conversely, private bank employees were found to have lower odds of LBP.

### Prevalence of LBP

The annual prevalence of LBP from this study is in line with studies conducted in Bhopal, India (51.8%) [12], Pakistan (52.4%) [13], and Ethiopia (55.4%) [35] among bank employees and Kavre, Nepal (52.0%) among construction workers [24]. This similarity in prevalence rate may be attributed to the common data collection tool and technique, and the similar occupation of participants. However, our study’s finding is higher than those reported in studies conducted among textile workers (35%) [27], motor bike riders (43.8%) [23] in Kathmandu, Nepal, and Bangladesh (36.6%) among bank employees [14]. Similarly, studies from eastern Nepal among the general population (71%) [26] and India (65.25%) among bank employees [11] reported a higher prevalence rate of LBP. These differences in the prevalence rate of LBP might be variations in data collection tools and measurement of the prevalence of LBP, as well as differences in the study period and sampling technique. The NMQ-E questionnaire is preferred over other tools to estimate the prevalence of LBP. Similarly, the difference in duration (i.e., past one month, past three months, or past 12 months) affects the prevalence of LBP. The longer the duration of the period, the higher the prevalence of LBP, since it takes a certain time to develop symptoms of LBP. Furthermore, the study conducted using probability sampling techniques will result in an accurate prevalence of LBP by ensuring a more representative sample from the population.

### Factors associated with LBP

In this study, females were more likely to have LBP than males. This finding is in agreement with studies conducted in Kathmandu [27] and Kavre [24], Nepal, Ethiopia [32], and the WHO Study on Global Aging and Adult Health [33]. Similarly, a systematic review and meta-analysis corroborate our finding; the odds of LBP were higher among females than males [28]. One plausible underlying reason for this might be that the threshold for pain is lower among women than men [49]. In addition, it might also be attributed to increased pain sensitivity among women, menstrual cycle fluctuations, and biological response to pregnancy and childbirth, as well as the physical stress of childbearing, that makes them more prone to LBP than men [50]. Moreover, differences in the prevalence of LBP among males and females may be due to sex differences related to gonadal steroid hormones, such as testosterone and estradiol modulate sensitivity to analgesia and pain [51]. This suggests the need for female-targeted ergonomic intervention at the workplace to address low back pain.

Employees with higher education have an increased likelihood of LBP compared to those with lower education. The plausible explanation for this association is that usually employees with higher education have managerial-level jobs, while people with lower education have lower-level jobs in the bank. Higher managerial jobs involve more mental stress and less physical work compared to lower-level jobs, thereby increasing the risk of LBP [52, 53]. This implies the need to implement ergonomic-friendly job duties for employees with higher education for the prevention of LBP.

However, a previous study from Ethiopia [31] and the WHO Study on Global Aging and Adult Health [33] showed that participants with low educational levels were more likely to have musculoskeletal disorders. Similarly, a systematic review and meta-analysis did not find a consistent association between education and the risk of LBP [54]. This incongruity of educational status with LBP could be attributed to heterogeneity in socio-economic status and the study population, suggesting further research using robust methodology, wider population and statistical control of socio-economic status while examining the association between education and LBP.

Like the previous studies from Ethiopia [31, 32], employees experiencing work-related stress were more likely to suffer from LBP compared to their counterparts. The underlying causal linkage may be that higher mental stress might amplify muscle tension and reduce micro-pause in the muscle activity, resulting in muscle fatigue even in the case of low loads due to continuous firing of low-threshold motor units [31, 55, 56]. Similarly, changes in nerves, hormones, and blood pressure due to work-related stress result in improved musculoskeletal co-activation, consequently increasing musculoskeletal system load and triggering LBP [57]. Moreover, the response of the central nervous system to job stress may augment painful sensation, thereby resulting in a higher prevalence of musculoskeletal disorders, including LBP [58]. Besides emotional exhaustion, higher emotional demands might heighten the risk of musculoskeletal pain, including LBP [59]. This finding underscores an urgent need to manage job-related stress among employees to reduce their risk of developing LBP.

Similarly, married employees were less likely to have LBP than unmarried and separated employees, as supported by a study from Nepal [26] and Brazil [30]. The plausible explanation for this could be that the support of a spouse in maintaining physical and mental health might result in lower muscle stress and chronic pain [60]. Similarly, compared to unmarried people, married people have a better quality of life [61] and social support [62], which lowers their risk of LBP through buffering stress and providing emotional support. Nonetheless, single employees are at higher risk of developing LBP, which might be linked with their poor mental health status since they receive less social support than married people [30]. This implies the importance of social support and mental health well-being among employees to alleviate their suffering from LBP.

Additionally, employees from private banks had lower odds of LBP than those from public banks. In Nepal, private banks may follow workplace standards and ergonomic practices more strictly than government banks. Since the human resource (HR) department in the private bank is responsible for improving the productivity of employees, there is proper compliance with working hours, sitting hours, and break time. These factors might put private bank employees at lower risk of LBP. In addition, there may be underreporting of LBP due to fear of getting tagged as ill. In contrast, there is only provision of personal management based on existing laws and regulations, and poor implementation of provisions related to occupational safety and health of the Labor Act 2017 in the public banks [63]. As a result, they might have increased odds of developing LBP. This suggests the need for proper implementation of occupational safety and health-related provisions and the development of an ergonomically friendly workplace.

Prolonged sitting (> 6 h daily) was prevalent (56.6%), in this study. However, it was not associated with LBP in the final model. This may reflect the homogeneous sedentary nature of banking work in our sample. However, we did not measure total weekly working hours, which might provide a more nuanced measure of occupational exposure-a consideration for future research.

According to the logistic regression modeling, gender, marital status, work-related stress, educational level, and type of bank were significantly associated with LBP. However, we used Mean Decrease in Accuracy (MDA) and Mean Decrease in Gini (MDG) to identify the most influential factors associated with LBP. Based on MDA and MDG, work-related stress, marital status, and gender were found to be more important factors than others. In the resource-limited setting, we cannot address all risk factors at once to address LBP; hence, identifying the main risk factors will help to rationally allocate priority and resources for health and workplace interventions for LBP.

### Implications of the study

The findings of this study have important implications for programs aimed at reducing the burden of LPB. Variable importance, measured based on MDA and MDG, showed that work-related stress, marital status, and gender are strong predictors of LBP among bank employees. Specifically, addressing the work-related stress of employees through cognitive, behavioral, and ergonomic interventions might help to reduce the LBP. Similarly, job stress management training could greatly benefit workers by lowering their risk of developing LBP. Additionally, the consistent association between work-related stress and marital status with LBP emphasizes the urgent need to tailor interventions to promote the mental health of workers. More importantly, female employees’ vulnerability to LBP should be addressed through targeted ergonomic interventions.

### Limitations of the study

This study has certain limitations that cannot be overlooked. First, we measured the prevalence of LBP using a questionnaire; however, we did not assess the severity of LBP and functional disability. Similarly, we have included participants with at least 3 months of working experience in the bank; however, we calculated the 1-year prevalence of LBP, creating potential exposure misclassification. To assess this, we determined that majority of participants had > 1 year of banking experience, substantially reducing this concern. The retrospective nature of information required to assess the LBP may give rise to recall bias. Nonetheless, we used a validated questionnaire, and the recall period was restricted to the past 12 months. Although social desirability bias might occur while reporting LBP. We mitigated this by using the validated NMQ-E with a defined recall period, ensuring anonymity, and employing neutral interview techniques.

Furthermore, we did not collect specific job positions (e.g., teller, manager), which may confer distinct ergonomic and psychosocial risks not fully captured by education level. Additionally, while we measured daily seated hours, we did not capture total weekly working hours, potentially limiting precision in assessing dose-response relationships. These factors should be addressed in future research with detailed occupational characterizations.

## Conclusions

This study reported that over half of the bank employees in Pokhara have experienced LBP in the past 12 months. The proportion of bank employees experiencing lifetime prevalence of LBP was even higher, at 74.5%. Gender, educational status, work-related stress, marital status, and type of bank were associated with LBP. To reduce LBP, a multifaceted intervention to prevent job-related stress is recommended. Furthermore, while designing ergonomic interventions, gender and marital status should be considered to alleviate the burden of LBP.

## Data Availability

The datasets used and/or analyzed during the current study are available from the corresponding author on reasonable request.

## Abbreviations

AIC: Akaike Information Criterion
AOR: Adjusted Odds Ratio
BIC: Bayesian Information Criterion
CI: Confidence Interval
IRB: Institutional Review Board
IQR: Inter Quartile Range
LBP: Low Back Pain
LLR: Log Likelihood Ratio
MDA: Mean Decrease in Accuracy
MDG: Mean Decrease in Gini
NMQ-E: Extended Nordic Musculoskeletal Questionnaire
SD: Standard Deviation
UOR: Unadjusted Odds Ratio
WHO: World Health Organization
YLD: Years Lived with Disability
